# Novel Orally Active Analgesic and Anti-Inflammatory Cyclohexyl-*N*-Acylhydrazone Derivatives

**DOI:** 10.3390/molecules20023067

**Published:** 2015-02-12

**Authors:** Tiago Fernandes da Silva, Walfrido Bispo Júnior, Magna Suzana Alexandre-Moreira, Fanny Nascimento Costa, Carlos Eduardo da Silva Monteiro, Fabio Furlan Ferreira, Regina Cely Rodrigues Barroso, François Noël, Roberto Takashi Sudo, Gisele Zapata-Sudo, Lídia Moreira Lima, Eliezer J. Barreiro

**Affiliations:** 1 Laboratório de Avaliação e Síntese de Substâncias Bioativas (LASSBio^®^), Instituto Nacional de Ciência e Tecnologia de Fármacos e Medicamentos (INCT-INOFAR), Universidade Federal do Rio de Janeiro, CCS, Cidade Universitária, P.O. Box 68024, Rio de Janeiro-RJ 21941-971, Brazil; E-Mail: tiagosilfer@yahoo.com.br; 2 Programa de Pós-Graduação em Química, Instituto de Química, Universidade Federal do Rio de Janeiro, Rio de Janeiro-RJ 21941-909, Brazil; 3 LaFI—Laboratório de Farmacologia e Imunidade, Instituto de Ciências Biológicas e da Saúde, Universidade Federal de Alagoas, Maceió-AL 57072-900, Brazil; E-Mails: walfridobispojr@hotmail.com (W.B.J.); suzana.magna@gmail.com (M.S.A.-M.); 4 Centro de Ciências Naturais e Humanas (CCNH), Universidade Federal do ABC (UFABC) Av. dos Estados 5001, Santo André-SP 09210-580, Brazil; E-Mails: fannycosta@yahoo.com.br (F.N.C.); fabio.furlan@ufabc.edu.br (F.F.F.); 5 Physics Institute, UERJ, Rio de Janeiro-RJ 20550-900, Brazil; E-Mail: cely_barroso@hotmail.com; 6 Programa de Pesquisa em Desenvolvimento de Fármacos, Instituto de Ciências Biomédicas, Universidade Federal do Rio de Janeiro, Rio de Janeiro-RJ 21941-902, Brazil; E-Mails: cadusilvarj@ig.com.br (C.E.S.M.); fnoel@pharma.ufrj.br (F.N.); rtsudo@farmaco.ufrj.br (R.T.S.); gsudo@farmaco.ufrj.br (G.Z.-S.)

**Keywords:** molecular simplification, *N*-acylhydrazone, anti-inflammatory, neuropathic pain, privileged structure, adenosine receptor

## Abstract

The *N*-acylhydrazone (NAH) moiety is considered a privileged structure, being present in many compounds with diverse pharmacological activities. Among the activities attributed to NAH derivatives anti-inflammatory and analgesic ones are recurrent. As part of a research program aiming at the design of new analgesic and anti-inflammatory lead-candidates, a series of cyclohexyl-*N*-acylhydrazones **10**–**26** were structurally designed from molecular modification on the prototype LASSBio-294, representing a new class of cycloalkyl analogues. Compounds **10**–**26** and their conformationally restricted analogue **9** were synthetized and evaluated as analgesic and anti-inflammatory agents in classical pharmacologic protocols. The cyclohexyl-*N*-acylhydrazones **10**–**26** and the cyclohexenyl analogue **9** showed great anti-inflammatory and/or analgesic activities, but compound **13** stood out as a new prototype to treat acute and chronic painful states due to its important analgesic activity in a neuropathic pain model.

## 1. Introduction

Defined as a minimum common subunit present in the structure of several drug candidates, privileged structures are characterized by their ability to be recognized by plural and distinct receptors. This concept has been widely used in the literature for the design of kinase-protein and serine-protease inhibitors, and ligands of G-protein coupled receptors (GPCR) and ionic channels [1]. Recently, based on several works with bioactive *N*-acylhydrazone (NAH) derivatives [2,3,4,5,6,7], the privileged structure nature of this bioactive framework was proposed [5].

**Figure 1:**
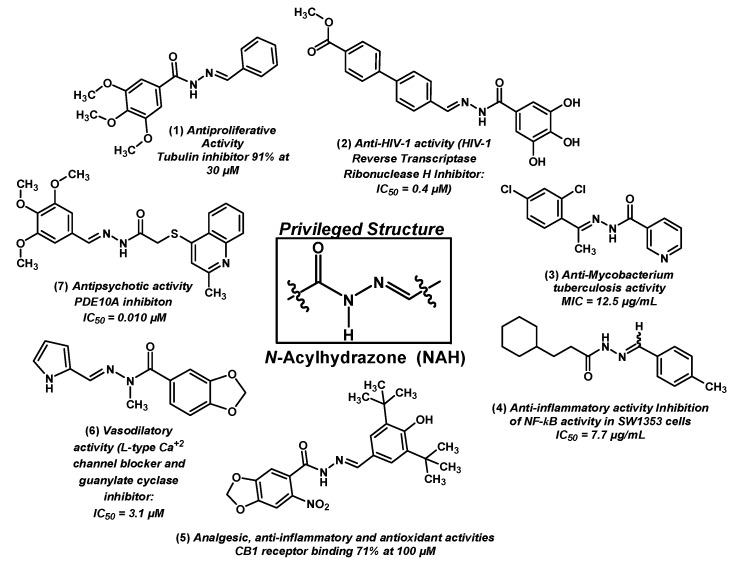
Examples of *N*-acylhydrazones with different mechanism of action and pharmacological activity.

Compounds **1**–**7** containing the NAH subunit have been described to possess antitumor (compound **1**) [6], antiviral (compound **2**) [8], antibacterial (compound **3**) [9], anti-inflammatory (compound **4**) [4], analgesic (compound **5**) [10,11], vasodilatory (compound **6**) [12] or antipsychotic (compound **7**) [13] activity (Figure 1).

**Figure 2:**
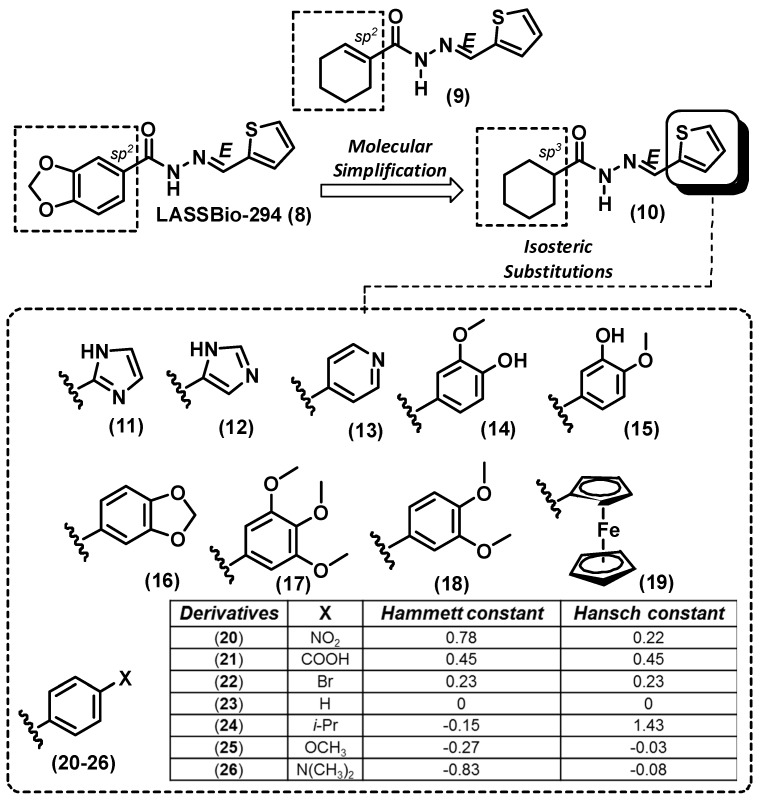
Design concept of novel cyclohexyl-*N*-acylhydrazones **10**–**26** from the prototype LASSBio-294 and its cyclohexenyl analogue **9**.

As part of a research program aiming at the design of new analgesic and anti-inflammatory lead-candidates, especially useful in the treatment of neuropathic pain, molecular changes in the prototype (*E*)-*N*'-((thiophen-2-yl)methylene)benzo[*d*][1,3]dioxole-5-carbohydrazide (LASSBio-294, **8**) were performed in order to improve its analgesic and anti-inflammatory profile. LASSBio-294 has been described as a potent positive inotropic agent with modest vasodilatory [14], analgesic and anti-inflammatory activities [15]. Structural modification in the derivative **8** was planned applying the concept of molecular simplification [16,17], resulting in the replacement of the 1,3-benzodioxole system by a cyclohexane moiety (Figure 2).

Considering the changes in the hybridization of the carbon atom linked to the carbonyl function in the cyclohexyl-*N*-acylhydrazone derivatives **10**–**26**, compared to the prototype LASSBio-294 (**8**), the cyclohexenyl analogue **9** was also designed. Aiming to construct a congeneric series, the 2-thienyl ring was replaced by several isosteric rings (compounds **10**–**19**) and the resulting phenyl nucleus was subsequently substituted by different monovalent substituents [18] (compounds **20**–**26**; Figure 2). The election of these substituents considered their electronic effects and their lipophilicity, in accordance with Hammett and Hansch parameters, as illustrated in Figure 2.

## 2. Results and Discussion

### 2.1. Chemistry

**Scheme 1:**
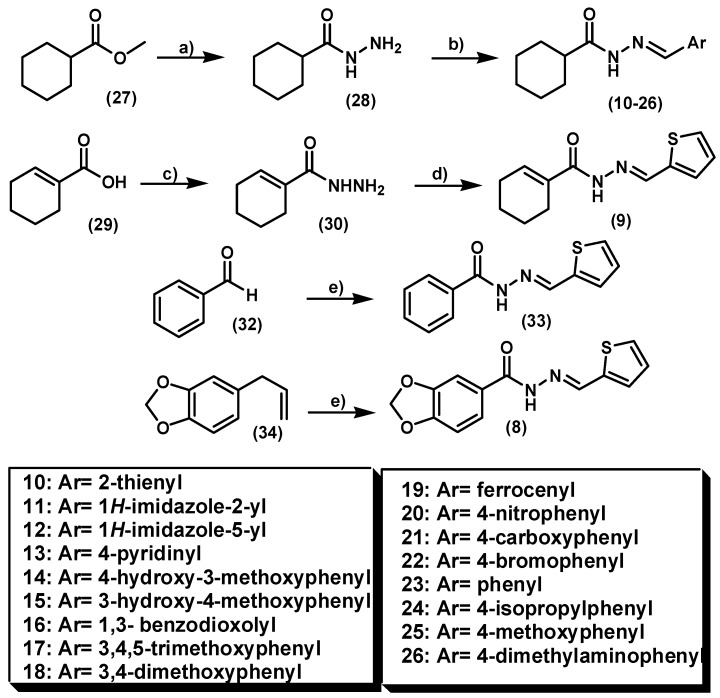
Synthesis route to NAH compounds.

The cyclohexyl-*N*-acylhydrazones **10**–**26** and the cyclohexenyl analogue **9** were synthesized using classical methodology as depicted in Scheme 1. Compounds were obtained in good yields by exploring a linear synthesis in two and three steps, based on functional group interconversion and acid-catalyzed condensation of hydrazide intermediates **28** or **30** with aldehydes, previously selected in accordance with the design concept illustrated in Figure 1. LASSBio-294 (**8**) and LASSBio-322 (**33**) [19,20] were used in order to compare the biological results with the analogues **10**–**26** and **9**; and to establish the contribution of 1,3-benzodioxole and phenyl subunits to the analgesic and anti-inflammatory activities.

*Reagents and conditions*: (**a**) NH_2_NH_2_·H_2_O 64%, EtOH, reflux, 48 h, 79%; (**b**) aromatic aldehyde, EtOH, room temperature, 30–90 min, 46%–99%; (**c**) (1) SOCl_2_, DMF (cat.), room temperature, 4 h; (2) NH_2_NH_2_·H_2_O 64%, CH_2_Cl_2_, 0 °C, 2 h; (**d**) 2-thiophenecarboxaldehyde, EtOH, room temperature, 30 min, 51%; (**e**) as previously described [19,20].

Considering the possibility that *N*-acylhydrazones (-CONHN=CHR) may exist as *syn/anti* amide conformers and either as *Z/E* geometrical isomers (-HC=N-) [21], a careful analysis of the ^1^H-NMR spectra of the compounds was performed.

**Figure 3:**
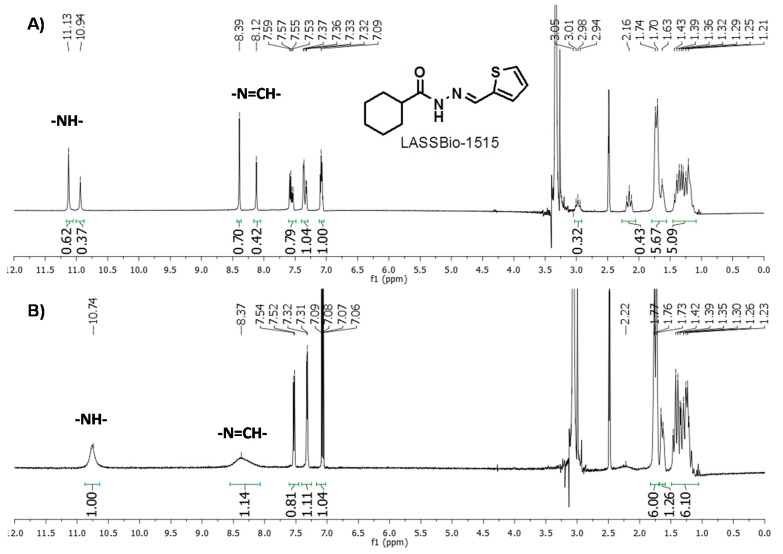
^1^H-NMR (300 MHz) spectra of the NAH derivative LASSBio-1515 (**10**) in DMSO*d_6_* at 25 °C (**A**) and 90 °C (**B**).

The ^1^H-NMR spectra of cyclohexyl-*N*-acylhydrazone derivative **10**, recorded at room temperature using solvents with different polarity (*i.e.*, DMSO-*d*_6_ and CDCl_3_), showed duplication of the singlet signals relative to amide and imine hydrogens (-CONHN=CH-) at 11 ppm in DMSO-*d_6_*. Curiously, the analysis of the same sample by reversed-phased HPLC, using different mobile phases, revealed only one peak at the chromatogram, indicating the presence of only one species. These data indicate that the mixture found in the ^1^H-NMR analysis does not correspond to possible geometric isomers (*Z* and *E*) of imine group (-N=CH-). In order to completely discard the possibility of diastereoisomers and to identify the possible presence of conformers of the amide bond, the ^1^H-NMR spectrum (300 MHz) of LASSBio-1515 (**10)** was recorded at 90 °C. This spectrum showed coalescence of the duplicated singlet signals seen in the ^1^H-NMR spectrum at room temperature (Figure 3). These results allowed us to discard the diastereoisomer possibility and reinforced the existence of conformers.

**Figure 4:**
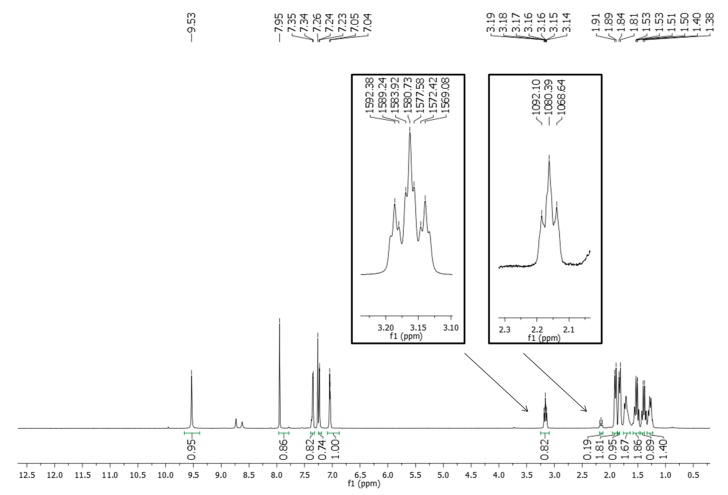
^1^H-NMR (500 MHz, CDCl_3_) spectrum of NAH derivative **10** with *J*-coupling of the tertiary hydrogen of the cyclohexyl functionalized system.

The possibility of chair conformers was also investigated by examining the *JJ*-coupling in the 500 MHz ^1^H-NMR of LASSBio-1515 (**10**). It was clear that both duplicated signals relative to the tertiary hydrogen had H-coupling constants of approximately 11 Hz (Figure 4). Data in the literature was found for axial-axial H-coupling [_1_H_axial_-_2_H_axial_ or _1_H_axial_-_6_H_axial_] of cyclohexyl derivatives, *J*_1Ha/2Ha_ or *J*_1-Ha/6Ha_ of around 8 to 14 Hz, while *J*_1He/2Ha_ or *J*_1-He/6Ha_ [_1_H_equatorial_-_2_H_axial_ or _1_H_equatoria_l-_6_H_axial_] have *J*-couplings below 5 Hz [22]. Therefore, the result for LASSBio-1515 (**10**) indicated that the mixtures of conformers at ^1^H-NMR are amide rotamers and not chair conformers. This type of rotamers was previously reported for NAH compounds [21,23,24]. The same ^1^H-NMR spectra analysis performed with compounds **8**, **9** and **33**, revealed that the presence of rotamers seems to be dependent of the hybridization nature of carbon linked to the carbonyl-amide group. The rotamers were not seen for the cyclohexenyl derivative **9** or even for phenyl analogue **33** and LASSBio-294 (**8**).

**Figure 5:**
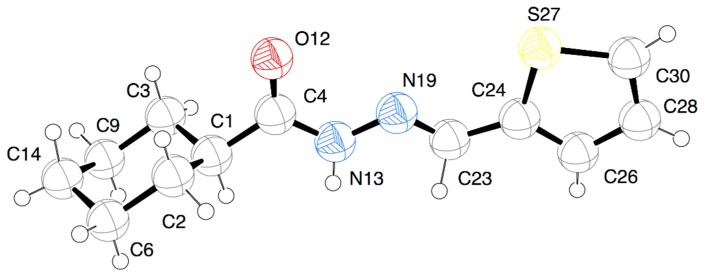
View of representative NAH derivative LASSBio-1515 (**10**) by X-ray powder diffraction Red = oxygen (O); blue = nitrogen (N); yellow = sulfur (S), black = carbon (C); and white = hydrogen (H).

Aiming to determine unambiguously the relative configuration and conformation of the cyclohexyl-*N*-acylhydrazone derivative **10** X-ray powder diffraction was performed [25]. The results indicated that in the solid phase the compound is an *E* diastereoisomer (-N=CH-), having equatorial substitution of the cyclohexyl ring, with an antiperiplanar conformation of the hydrogen amide and oxygen atom of the carbonyl group (Figure 5).

### 2.2. Pharmacological Activities

The anti-inflammatory and analgesic activities of the cyclohexyl-*N*-acylhydrazones **10**–**26** and the cyclohexenyl analogue **9** were determined using classical murine models, exemplified by carrageenan-induced peritonitis, acetic acid-induced writhing and formalin-induced pain [26,27,28]. The results were compared with the standard drug indomethacin and with the prototype LASSBio-294 (**8**) and its simplified analogue LASSBio-382 (**33**). All compounds were tested by oral administration using a screening dose of 100 µmol/Kg.

As illustrated in Table 1 the replacement of the 1,3-benzodiozole system (compound **8**) by a cyclohexyl subunit (compound **10**) resulted in the improvement of the anti-inflammatory and analgesic activities. The conformationally restricted cyclohexenyl analogue **9** was significantly less active than the cyclohexyl derivative **10** in the carrageenan-induced peritonitis model and showed similar antihipernociceptive profile in the acetic acid-induced writhing test (Table 1). The attempts to replace the 2-thienyl ring (in **10**) by isosteric ones, like a 4-pyridinyl (compound **13**), 1*H*-imidazole-2-yl (compound **11**) and the 1*H*-imidazole-5-yl regioisomer (compound **12**) resulted in loss of activity in these models for compounds **11** and **12**, while derivative **13** showed a similar pharmacological profile as the lead **10** (Table 1) The isosteric replacement of the 2-thienyl unit in **10** by a phenyl group (compound **23**) decreased the anti-inflammatory activity, although the analgesic profile in the writhing test was preserved (Table 1). The substitution of the phenyl ring of compound **23** by monovalent isosteric groups (e.g., **10**–**26**) improves the anti-inflammatory and analgesic activities, with the regioisosmers **14** and **15** and the 4-dimethylamine (**26**) and 4-bromo (**22**) derivatives standing out. The ferrocenyl derivative **19**, designed in accordance with literature reports showing its bioisosteric relationship with the phenyl group [29], showed an improvement in the analgesic profile (Table 1) in the acetic acid-induced writhing test and also in the 1st phase of the formalin model.

In the formalin test, a neurogenic phase associated with the direct effect on sensory C fibers (1st phase) is followed by an inflammatory response related to analgesic mediators (2nd phase) [30]. With this in mind, compounds **19**, **22** and **26**, which were active in the neurogenic phase of the formalin test, were selected to be studied in the hot plate test [31]. These compounds were inactive in this model (data not shown), discarding the hypothesis of a central antinociceptive activity and reinforcing the peripheral analgesic profile of these bioactive *N*-acylhydrazones.

Considering recent works reporting the ability of the prototype **8** (LASSBio-294) to be a ligand of the adenosine receptor, subtype A_2A_ [32], the cyclohexyl-*N*-acylhydrazones **10**–**26** and the cyclohexenyl analogue **9** were investigated using a specific binding assay for the A_2A_ receptor (Table 1). As shown in Table 1, LASSBio-294 presented a *K*i value of 8.2 µM. However, its simplified analogues **9**, **10**–**26** and **33** were unable to bind to the A_2A_ adenosine receptor, suggesting the pharmacophoric characteristic of 1,3-benzodiozole scaffold.

Bearing in mind that neuropathic pain represents a great challenge, and considering that its treatment needs more specific and effective drugs, the analgesic activity of the cyclohexyl-*N*-acylhydrazones in a murine neurophatic pain model was investigated. For this purpose, the spinal nerve ligation (SNL) model in rats [33] was selected, since involvement of inflammatory and analgesic components has been reported in this neuropathic pain model [34]. Compound **19** (LASSBio-1517), which presented a high analgesic activity in the acetic acid induced writhing test, **17** (LASSBio-1513) and **13** (LASSBio-1514), that showed comparable analgesic and anti-inflammatory activities (Table 1), were evaluated in the SNL model. 

**Table 1:** Effect of *N*-acylhydrazones (10 or 30 µM in binding assay and 100 µmol/kg p.o. in the *in vivo* assays), dipyrone or indomethacin (100 µmol/kg, p.o *in vivo* assays).

Biological Tests							
	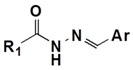		Binding A2a	Carrageenan-induced peritonitis	Acetic acid-induced writhing test	Formalin-induced pain test	
Compounds	(R_1_)	(Ar)	% of inhibition ^a^	% of inhibition ^b^	% of inhibition ^b^	Phase I % of inhibition ^b^	Phase II % of inhibition ^b^
**Dipyrone or Indomethacin**			-	65.1 ± 4.0 ** dipyrone	82.5 ± 6.2 ** indomethacin	23.5 ± 7.9 indomethacin	57.4 ± 5.8 ** indomethacin
**(8) ** **LASSBio-294**	1,3-benzodioxolyl	2-thienyl	(Ki = 8.2µM)	55.3 ± 4.8 **	68.2 ± 13.6 **	37.4 ± 4.9	42.0 ± 4.7 **
**(33) ** **LASSBio-382**	phenyl	2-thienyl	17% at 10 µM	40.3 ± 2.3 **	57.2 ± 8.5 **	12.3 ± 6.1	23.2 ± 4.1
**(9) ** **LASSBio-1780**	cyclohexenyl	2-thienyl	7% at 10 µM	21.4 ± 5.4 **	77.3 ± 3.2 **	-	-
**(10) ** **LASSBio-1515**	cyclohexyl	2-thienyl	13% at 30 µM	79.2 ± 2.7 **	87.9 ± 3.4 **	32.9 ± 11.5	61.5 ± 2.9 **
**(11) ** **LASSBio-1600**	cyclohexyl	1H-imidazole-5-yl	36% at 10 µM	39.8 ± 4.9 **	20.9 ± 5.7 *	5.2 ± 3.2	15.4 ± 10.6
**(12) ** **LASSBio-1602**	cyclohexyl	1H-imidazole-2-yl	0% at 10 µM	37.6 ± 1.9 **	69.2 ± 7.9 **	14.2 ± 5.8	5.7 ± 2.4
**(13) ** **LASSBio-1514**	cyclohexyl	4-pyridinyl	0% at 10 µM	81.9 ± 2.2 **	65.7 ± 9.5 **	36.9 ± 8.5	43.0 ± 5.7 **
**(14) ** **LASSBio-1688**	cyclohexyl	3-hydroxy-4-methoxyphenyl	15% at 30 µM	74.3 ± 4.4 **	90.7 ± 2.5 **	36.9 ± 8.5	38.3 ± 5.8 **
**(15) ** **LASSBio-1689**	cyclohexyl	4-hydroxy-3-methoxyphenyl	-	68.2 ± 4.9 **	55.6 ± 5.3 **	31.5 ± 8.3	63.1 ± 7.7 **
**(16) ** **LASSBio-1691**	cyclohexyl	1,3- benzodioxolyl	33% at 30 µM	64.7 ± 3.0 **	56.0 ± 11.9 **	31.3 ± 6.7	25.6 ± 5.9
**(17) ** **LASSBio-1513**	cyclohexyl	3,4,5-trimethoxyphenyl	10% at 30 µM	76.3 ± 3.8 **	78.3 ± 8.4 **	26.9 ± 5.8	38.5 ± 6.8 **
**(18) ** **LASSBio-1509**	cyclohexyl	3,4-dimethoxyphenyl	9% at 10 µM	63.7± 3.2 **	67.3 ± 8.5 **	13.1 ± 7.8	75.97 ± 4.4 **
**(19) ** **LASSBio-1517**	cyclohexyl	ferrocenyl	5% at 30 µM	46.9 ± 6.7 **	97.6 ± 0.9 **	47.3 ± 4.7 **	27.1 ± 12.0
**(20) ** **LASSBio-1603**	cyclohexyl	4-nitrophenyl	0% at 10 µM	42.8 ± 1.9 **	47.6 ± 7.0 **	31.3 ± 8.6	25.9 ± 6.9
**(21) ** **LASSBio-1516**	cyclohexyl	4-carboxylic-phenyl	0%, at 10 µM	58.6 ± 5.3 **	87.4 ± 6.0 **	17.5 ± 5.9	54.7 ± 6.9 **
**(22) ** **LASSBio-1511**	cyclohexyl	4-bromophenyl	3% at 10 µM	70.7 ± 3.1 **	62.2 ± 2.1 **	53.4 ± 11.1 **	77.0 ± 3.9 **
**(23) ** **LASSBio-1601**	cyclohexyl	phenyl	8% at 10 µM	28.7 ± 4.4 **	57.3 ± 9.5 **	20.3± 8.4	NA
**(24) ** **LASSBio-1508**	cyclohexyl	4-isopropylphenyl	12% at 10 µM	78.6 ± 3.3 **	64.9 ± 3.3 **	13.4 ± 8.2	38.5 ± 6.8 *
**(25) ** **LASSBio-1510**	cyclohexyl	4-methoxyphenyl	0% at 10 µM	67.4 ± 4.9 **	86.3 ± 4.0 **	30.1 ± 6.0	70.1 ± 5.3 **
**(26) ** **LASSBio-1512**	cyclohexyl	4-dimethylaminophenyl	0% at 10 µM	63.9 ± 4.0 **	68.3 ± 13.4 **	38.9 ± 13.1 *	54.4 ± 4.9 **

^a^ The % inhibition of specific binding displaced by 10 or 30µM competing compound. ^b^ The asterisks denote the significance levels in comparison with control groups (* *p* < 0.05, ** *p* < 0.01 at ANOVA). NA: not active.

**Figure 6:**
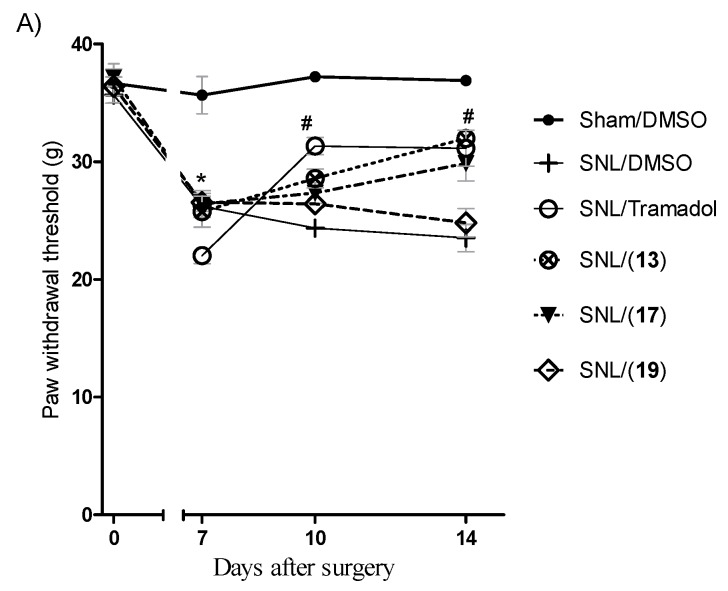

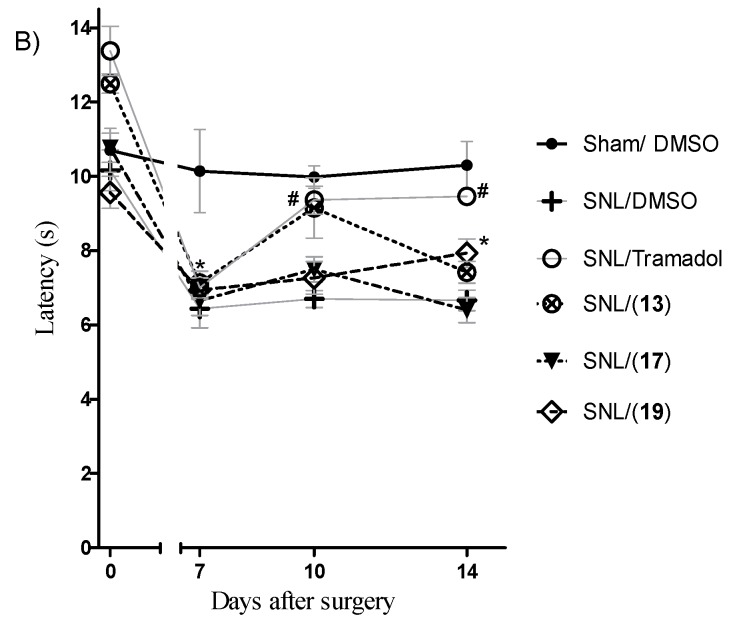
Spinal nerve ligation (SNL) model in rats. (**A**) Treatment of mechanical allodynia for 7 days orally at a dose of 100 μmol·kg^−1^ (tramadol 33.3 μmol·kg^−1^), *n* = 6 animals. (**B**) Treatment of thermal hyperalgesia for 7 days orally at a dose of 100 μmol·kg^−1^ (tramadol 33.3 μmol·kg^−1^), *n* = 6 animals. * *p* < 0.05 *versus* day 0. ^#^ *p* < 0.05 *versus* day 7. One-way ANOVA, followed by the Newman–Keuls test. *p* values lower than 0.05 were considered significant.

As demonstrated in Figure 6A, oral treatment for seven days with 100 µmol/kg of compounds **17** (LASSBio-1513) and **13** (LASSBio-1514) prevented the mechanical allodynia, while **19** (LASSBio-1517) was inactive. Derivative **13** (LASSBio-1514) was also able to increase the latency time in the thermal hyperalgesia component of the SNL model, revealing an important antihyperalgesic activity (Figure 6B). The better profile observed with acylhydrazones **13** and **17** on SNL model may be explained by their associative analgesic and anti-inflammatory activities (Table 1). Although the results presented in this study did not clarify the mechanism of action of these bioactive cyclohexyl-*N*-acylhydrazones, the results suggest that these compounds could be considered as new lead-candidates to treat acute and chronic painful states.

## 3. Experimental Section

### 3.1. Chemistry

#### 3.1.1. General Methods

NMR spectra were determined in deuterated chloroform or dimethyl sulfoxide containing ca. 1% tetramethylsilane as an internal standard, using a 200/50 MHz Bruker DPX-200, 250/62.5 MHz Bruker DPX-250, 400/100 MHz Varian 400-Mr, 300/75 MHz Varian Unity-300 and 500/125 MHz Varian VNMRSYS-500 spectrometer. The progress of all reactions was monitored by thin layer chromatography, which was performed on 2.0 cm × 6.0 cm aluminum sheets pre-coated with silica gel 60 (HF-254, Merck) to a thickness of 0.25 mm, or by infrared (IR) spectra obtained with Nicolet-550 Magna spectrophotometer by using potassium plates. The developed chromatograms were viewed under ultraviolet light at 254 nm. Merck silica gel (70–230 mesh) was used for column chromatography. Elemental analyses were carried out on a Thermo Scientific Flash EA 1112 Series CHN-Analyzer. Melting points were determined with a Quimis 340 apparatus and are uncorrected. All described products showed ^1^H and ^13^C NMR spectra according to the assigned structures. All organic solutions were dried over anhydrous sodium sulfate and all organic solvents were removed under reduced pressure in rotatory evaporator. HPLC for purity determinations were conducted using Shimadzu LC-20AD with a SHIM-PACK CLC-ODS analytical column (4.6 mm × 6250 mm) or Kromasil 100-5C18 (4.6 mm × 6250 mm) and a Shimadzu SPD-M20A detector at 254 nm wavelength. The solvent system for HPLC purity analyses was 70:30 acetonitrile:phosphate buffer solution at pH 7. The isocratic HPLC mode was used, and the flow rate was 1.0 mL/min.

#### 3.1.2. General Procedure for the Preparation of the 3,4-Methylenedioxybenzoylacylhydrazone LASSBio-294 (**8**) and the Methylenedioxybenzoylacylhydrazone LASSBio-382 (**33**)

The methodology used in the synthesis of compounds **8** (LASSBio-294) and **33** (LASSBio-332) was previously reported by our research group [17,19,20] and was reproduced in this work.

#### 3.1.3. Procedure for the Synthesis of Cyclohex-1-enecarbohydrazide Intermediate **30**

A solution of cyclohex-1-enecarboxylic acid (**29**, 0.3 g, 2.38 mmol) and thionyl chloride (2 mL) in the presence of one drop of DMF was stirred at room temperature for 4 h until reaction completion. The solution was concentrated to dryness under reduced pressure to obtain a solid residue, which was solubilized in dichloromethane (10 mL) for the next step without further purification. To a reaction flask containing 80% hydrazine monohydrate (1.15 mL, 23.80 mmol) in dichloromethane (10 mL) was added dropwise the first solution at 0 °C for 2 h. At the end of the reaction, the solution was concentrated under reduced pressure. The solid **30** formed was filtered off and washed with *n*-hexane. 

#### 3.1.4. Procedure for the Synthesis *of* (*E*)-*N*'-(Thiophen-2-ylmethylene)cyclohex-1-enecarbohydrazide (LASSBio-1780, **9**)

The cyclohex-1-enecarbohydrazide **30** previously obtained was immediately used without further purification or characterization. After solubilization in ethanol (21 mL) thiophene-2-carboxaldehyde (2.38 mmol) was added. The mixture was stirred at room temperature for 30 min, until TLC indicated the end of the reaction. The resulting suspension was poured onto ice and the resulting precipitate was filtered out and washed with *n*-hexane. Compound **9** (LASSBio-1780) was obtained as a yellow solid in 51% yield, m.p. 201–203 °C. ^1^H-NMR (400 MHz) DMSO-*d*_6_ (ppm): 11.17 (s, 1H, CONH), 8.55 (s, 1H, N=CH), 7.61 (d, 1H, *J* = 5 Hz), 7.38 (d, 1H, *J* = 5 Hz), 7.13 (dd, 1H, *J* = 5 Hz, *J* = 2 Hz), 6.57 (m, 1H), 2.21–1.17 (m, 4H, cyclohexenyl), 1.71–1.21 (m, 4H, cyclohexenyl). ^13^C-NMR (50 MHz) DMSO-*d*_6_ (ppm): 164.45 (C=O), 141.77 (1C), 139.36 (1C), 133.16 (1C), 132.62 (C1), 130.27 (1C), 128.46 (1C), 127.72 (1C), 23–25 (2C), 21–22 (2C). IR (KBr)ν_max_ (cm^−1^): 3215 (NH); 2932 (CH_3_); 1660 (C=C); 1626 (C=O); 1596 (C=N). % purity = 96.93% by HPLC-C18 (acetonitrile/water 7:3) (Rt = 3.89 min).

#### 3.1.5. Procedure for the Preparation of Cyclohexanecarbohydrazide (**28**)

Hydrazine hydrate (64%, 1.36 mL, 28.13 mmol) was added to a solution of methyl cyclohexanecarboxylate (**27**, 1.00 g, 7.03 mmol) in absolute ethanol (5 mL). The reaction mixture was kept under reflux for 48 h, whenupon infrared (IR) spectroscopy indicated the end of the reaction. Next, the ethanol was almost totally evaporated under reduced pressure. Ice was added to the residue and the resulting precipitate was filtered out affording the title compound in 79% yield, as white crystals, m.p. 154–156 °C. ^1^H-NMR (200 MHz, DMSO-*d_6_*, TMS) (ppm): 8.85 (s, 1H, CONH), 4.04 (s, 2H, NH_2_), 2.01 (m, 1H, cyclohexyl), 1.65 (m, 5H, cyclohexyl), 1.35 (m, 5H, cyclohexyl). ^13^C-NMR (50 MHz, DMSO-*d_6_*, TMS) (ppm): 174.92 (NC=O), 42.35 (1C, cyclohexyl), 29.19 (2C, cyclohexyl), 25.43 (1C, cyclohexyl), 25.33 (2C, cyclohexyl). IR (KBr) ν (cm^−1^): 3311, 3195 (NH); 2930 (CH); 1629 (C=O).

#### 3.1.6. General Procedure for Preparation of Cyclohexylacylhydrazones **10**–**26**

The corresponding aromatic or heteroaromatic aldehyde (2.11 mmol) was added to a solution of cyclohexanecarbohydrazide (**28**, 0.3 g; 2.11 mmol) in absolute ethanol (21 mL). The mixture was stirred for 2 h at room temperature. At the end of the reaction, the volume of ethanol was partially concentrated at reduced pressure and the resulting mixture was poured into cold water. The precipitate was filtered out, dried under vacuum and then the solid was washed with *n*-hexane and/or recrystallized from ethanol to give the desired cyclohexyl-*N*-acylhydrazones **10**–**26**.

*(E)-N'-(Thiophen-2-ylmethylene)cyclohexanecarbohydrazide* (LASSBio-1515, **10**): LASSBio-1515 (**10**) was obtained as orange crystals in 92% yield by condensation of cyclohexane-carbohydrazide (**28**) with thiophene-2-carboxylaldehyde, m.p. 209–211 °C. The data for this compound is in agreement with previous reports [35]. ^1^H-NMR (300 MHz) DMSO-*d*_6_ (ppm): 10.74 (s, 1H, CONH), 8.37 (s, 1H, N=CH), 7.55 (d, 1H, *J* = 5 Hz), 7.33 (d, 1H, *J* = 5 Hz), 7.09 (dd, 1H, *J* = 5 Hz, *J* = 1 Hz), 1,77 (m, 1H), 1.76 (m, 5H, cyclohexyl), 1.39 (m, 5H, cyclohexyl). ^13^C-NMR (50 MHz) DMSO-*d*_6_ (ppm): 176.60 and 171.57 (NC=O), 141.18 (1C), 137.29 (HC=N), 130.30 (3C). IR (KBr) ν_max_ (cm^−1^): 3185 (NH); 2926 (CH_3_); 1656 (C=O); 1590 (C=N). % purity = 96.55% by HPLC-C18 (acetonitrile/water 7:3) (Rt = 4.75 min).

*(E)-N'-((1H-imidazol-2-yl)methylene)cyclohexanecarbohydrazide* (LASSBio-1602, **11**): LASSBio-1602 (**11**) was obtained as a white solid in 57% yield by condensation of cyclohexane-carbohydrazide (**28**) with 1*H*-imidazole-2-carbaldehyde, m.p. > 220 °C. ^1^H-NMR (200 MHz) DMSO-*d*_6_ (ppm): 12.60 and 12.35 (s, NH, imidazole), 11.18 and 10.95 (s, 1H, CONH), 8.09 and 7.87 (s, 1H , HC=N), 7.21 (m, 2H), 2.86 and 2.19 (m, 1H), 1.72 (m, 5H, cyclohexyl), 1.32 (m, 5H, cyclohexyl). ^13^C-NMR (50 MHz) DMSO-*d*_6_ (ppm): 177.75 and 172.54 (NC=O), 143.08 (HC=N), 134.46 (1C), 137.87 (2C), 43.26 (1C), 29.39 (2C), 25.75 (3C). IR (KBr) ν_max_ (cm^−1^): 3188 (NH); 2925 (CH_3_); 1649 (C=O). Anal. Calcd. for C_11_H_16_N_4_O: C, 60.26; H, 6.90; N, 25.55. Found: C, 59.97; H, 6.95; N, 25.13.). % purity = 98.49% by HPLC-C18 (acetonitrile/water 6:4) (Rt = 2.98 min).

*(E)-N'-((1H-Imidazol-5-yl)methylene)cyclohexanecarbohydrazide* (LASSBio-1600, **12**): LASSBio-1600 (**12**) was obtained as a white solid in 46% yield by condensation of cyclohexane-carbohydrazide (**28**) with 1*H*-imidazole-5-carbaldehyde, m.p. > 220 °C. ^1^H-NMR (200 MHz) DMSO-*d*_6_ (ppm): 12.36 (NH, imidazole), 11.05 and 10.80 (s, 1H, CONH), 8.13 and 7.91 (s, 1H, HC=N), 7.69 (s, 1H), 7,42 (s, 1H), 3,13 and 2,19 (m, 1H), 1.72 (m, 5H, cyclohexyl), 1.32 (m, 5H, cyclohexyl).^13^C-NMR (50 MHz) DMSO-*d*_6_ (ppm): 171.83 and 177.20 (NC=O), 137.51 (HC=N), 136.23 (1C), 132.14 (1C), 121.44 (1C), 43.34 (1C), 29.52 (2C), 25.82 (3C). IR (KBr) ν_max_ (cm^−1^): 3188 (NH); 2925 (CH_3_); 1649 (C=O). Anal. Calcd. for C_11_H_16_N_4_O: C, 60.26; H, 6.90; N, 25.55. Found: C, 60.01; H, 7.23; N, 26.00.

*(E)-N'-(Pyridin-4-ylmethylene)cyclohexanecarbohydrazide* (LASSBio-1514, **13**): LASSBio-1514 (**13**) was obtained in 79% yield as yellow crystals by condensation of cyclohexane-carbohydrazide (**28**) with 4-pyridinecarboxaldehyde, m.p. 151–153 °C. The compound’s data was in agreement with previous reports [35]. ^1^H-NMR (200 MHz) DMSO-*d*_6_ (ppm): 11.55 and 11.46 (s, 1H, CONH), 8.63 (m, 2H), 8.34 and 8.17(s, 1H, N=CH), 7.59 (m, 2H), 3.10 and 2.19 (m, 1H), 1.72 (m, 5H, cyclohexyl), 1.32 (m, 5H, cyclohexyl). ^13^C-NMR (50 MHz) DMSO-*d*_6_ (ppm): 177.28 and 172.08 (NC=O), 150.22 (2C), 143.46 and 139.82 (HC=N), 141.66 (1C), 120.84 (2C), 43.40 (1C), 28.89 (2C), 25.31 (3C). IR (KBr) ν_max_ (cm^−1^): 3478 (NH); 2934 (CH_3_); 1664 (C=O); 1599 (C=N); 1397 (Py). Anal. Calcd. for C_13_H_17_N_3_O.H_2_O: C, 62.63; H, 7.68; N, 16.85. Found: C, 62.22; H, 7.61; N, 16,69.

*(E)-N'-(4-Hydroxy-3-methoxybenzylidene)cyclohexanecarbohydrazide* (LASSBio-1689, **14**): LASSBio-1689 (**14**) was obtained in 90% yield as white crystals by condensation of cyclohexane-carbohydrazide (**28**) with 4-hydroxy-3-methoxybenzaldehyde, m.p. 198–200 °C. ^1^H-NMR (200 MHz) DMSO-*d*_6_ (ppm): 11.43 and 11.10 (s, 1H, CONH), 9.47 (s, 1H, OH), 8.00 (s, 1H, N=CH), 7.24 (d, 1H, *J* = 8 Hz), 7.04 (d, 1H, *J* = 8 Hz), 6.82 (d, 1H, *J* = 8 Hz), 3.80 (s, 3H, CH_3_), 3.10 and 2.19 (m, 1H), 1.72 (m, 5H, cyclohexyl), 1.32 (m, 5H, cyclohexyl).^13^C-NMR (50 MHz) DMSO-*d*_6_ (ppm): 176.66 and 171.44 (NC=O), 148.76 (1C), 148.02 (1C), 146.50 and 142.53 (N=CH), 125.90 (1C), 121.81 (1C), 115.63 (1C), 109.39 (1C), 55.58 (1C), 42.89 (1C), 29.09 (2C), 25.73 (3C). IR (KBr) ν_max_ (cm^−1^): 3528–2850 (OH); 3528 (NH); 2930 (CH_3_); 1641 (C=O); 1281, 1204 (OCH_3_). % purity = 99.87% by HPLC-C18 (acetonitrile/water 7:3) (Rt = 3.48 min).

*(E)-N'-(3-Hydroxy-4-methoxybenzylidene)cyclohexanecarbohydrazide* (LASSBio-1688, **15**): LASSBio-1688 (**15**) was obtained in 92% yield as white crystals by condensation of cyclohexane-carbohydrazide (**28**) with 3-hydroxy-4-methoxybenzaldehyde, m.p. 192–194 °C. ^1^H-NMR (200 MHz) DMSO-*d*_6_ (ppm): 11.51 and 11.11 (s, 1H, CONH), 9.29 (s, 1H, OH), 8.00 (s, 1H, N=CH), 7.19 (d, 1H, *J* = 8 Hz), 6.99 (d, 2H, *J* = 8 Hz), 3.80 (s, 3H, CH_3_), 3.10 and 2.19(m, 1H), 1.72 (m, 5H, cyclohexyl), 1.32 (m, 5H, cyclohexyl). ^13^C-NMR (50 MHz) DMSO-*d*_6_ (ppm): 176.71 and 171.48 (NC=O), 149.61 (1C), 146.83 (N=CH), 142.59 (1C), 127.33 (1C), 120.03 (1C), 112.30 (1C), 111.90 (1C), 55.62 (1C), 43.91 (1C), 29.08 (2C), 25.41 (3C). IR (KBr) ν_max_ (cm^−1^): 3234–2850 (OH); 3234 (NH); 2930 (CH_3_); 1656 (C=O) 1259, 1204 (OCH_3_). % purity = 98.51% by HPLC-C18 (acetonitrile/water 6:4) (Rt = 3.97 min).

*(E)-N'-(Benzo[d][1,3]dioxol-5-ylmethylene)cyclohexanecarbohydrazide* (LASSBio-1691, **16**): LASSBio-1691 (**16**) was obtained in 98% yield as a white solid by condensation of cyclohexane-carbohydrazide (**28**) with benzo[*d*][1,3]dioxole-5-carbaldehyde, m.p. 206–208 °C. The compound’s data was in agreement with previous reports [36]. ^1^H-NMR (200 MHz) DMSO-*d*_6_ (ppm): 11.18 and 10.98 (s, 1H, CONH), 8.08 and 7.84 (s, 1H, N=CH), 7.22 (s, 1H), 7.09 (d, 1H, *J* = 8 Hz), 6.94 (d, 1H, *J* = 8 Hz), 6.11 (s, 2H, CH_2_), 3.10 and 2.19 (m, 1H), 1.72 (m, 5H, cyclohexyl), 1.32 (m, 5H, cyclohexyl). ^13^C-NMR (50 MHz) DMSO-*d*_6_ (ppm): 177.31 and 172.07 (NC=O), 149.41 (1C), 148.47 (1C), 146.28 and 142.54 (N=CH), 129.42 (1C), 123.53 (1C), 108.94 (1C), 105.58 (1C), 102.01 (1C, O-CH2-O), 43.40 (1C), 29.56 (2C), 25,83 (3C). IR (KBr) ν_max_ (cm^−1^): 3228 (NH); 2942 (CH_3_); 1659 (C=O) 1247 (OCH_3_). % purity = 95.33% by HPLC-C18 (acetonitrile/water 7:3) (Rt = 4.68 min).

*(E)-N'-(3,4,5-Trimethoxybenzylidene)cyclohexanecarbohydrazide* (LASSBio-1513, **17**): LASSBio-1513 (**17**) was obtained in 99% yield as a white solid by condensation of cyclohexane-carbohydrazide (**28**) with 3,4,5-trimethoxybenzaldehyde, m.p. 215–217 °C. ^1^H-NMR (200 MHz) DMSO-*d*_6_ (ppm): 11.27 and 11.11 (s, 1H, CONH), 8.65 and 8.10 (s, 1H, N=CH), 7.22 (s, 2H), 3.81 (s, 9H), 3.10 and 2.19 (m, 1H), 1.72 (m, 5H, cyclohexyl), 1.32 (m, 5H, cyclohexyl). ^13^C-NMR (50 MHz) DMSO-*d*_6_ (ppm): 177.42 and 172.21 (NC=O), 153.69 (2C), 146.49 and 142.24 (HC=N), 139.60 (1C), 130.60 (1C), 106.21 (2C), 60.71 (1C), 56.49 (2C), 43.40 (1C), 29.56 (2C), 25.73 (3C). IR (KBr) ν_max_ (cm^−1^): 3230 (NH); 2925 (CH_3_); 1660 (C=O); 1236 (O-CH_3_). Anal. Calcd. for C_17_H_24_N_2_O_4_: C, 63.73; H, 7.55; N, 8.74. Found: C, 63.54; H, 7.42; N, 8.51.

*(E)-N'-(3,4-Dimethoxybenzylidene)cyclohexanecarbohydrazide* (LASSBio-1509, **18**): LASSBio-1509 (**18**) was obtained in 94% yield as a white solid by condensation of cyclohexane-carbohydrazide (**28**) with 3,4-dimethoxybenzaldehyde, m.p. 193–195 °C. ^1^H-NMR (200 MHz) DMSO-*d*_6_ (ppm): 11.15 and 10.98 (s, 1H, CONH), 8.09 and 7.89 (s, 1H, N=CH), 7.27 (d, 1H, *J* = 2 Hz), 7.14 (dd, 1H, *J* = 8 Hz, *J* = 2 Hz), 6.99 (d, 1H, *J* = 8 Hz), 3.82 (s, 6H, OCH_3_), 3.10 na 2.19 (m,1H), 1.72 (m, 5H, cyclohexyl), 1.32 (m, 5H, cyclohexyl). ^13^C-NMR (50 MHz) DMSO-*d*_6_ (ppm): 177.24 and 172.00 (NC=O), 161.08 and 152.10 (1C), 150.90 (1C), 146.47 and 142.82 (HC=N), 127.79 (1C), 122.04 (1C), 112.14 (1C), 108.83 (1C), 55.98 (2C), 43.40 (1C), 29.57 and 28.92 (2C), 25.97 (3C). IR (KBr) ν_max_ (cm^−1^): 3223 (NH); 2926 (CH_3_); 1659 (C=O); 1603 (C=N); 1266, 1210(C-O). Anal. Calcd. for C_16_H_22_N_2_O_3_: C, 66.18; H, 7.64; N, 9.65. Found: C: 66.17; H: 7.48; N: 9.40.

*(E)-N'-Ferrocenylcyclohexanecarbohydrazide* (LASSBio-1517, **19**): LASSBio-1517 (**19**) was obtained in 99% yield as a brown solid by condensation of cyclohexane-carbohydrazide (**28**) with ferrocenecarboxaldehyde, m.p. > 220 °C. ^1^H-NMR (200 MHz) DMSO-*d*_6_ (ppm): 11.01 and 10.72 (s, 1H, CONH), 7.98 and 7.83 (s, 1H, N=CH), 4.57 (s, 2H), 4.42 (s, 2H), 4.19 (s, 5H), 2.86 and 2.19 (m, 1H), 1.72 (m, 5H, cyclohexyl), 1.32 (m, 5H, cyclohexyl). ^13^C-NMR (50 MHz) DMSO-*d*_6_ (ppm): 176.09 (NC=O), 142.67 (HC=N), 79.42 (1C), 69.83 (2C), 68.79 (5C), 67.28 (2C), 43.40 (1C), 29.51 (2C), 25.89 (3C). IR (KBr) ν_max_ (cm^−1^): 3189 (NH); 2929 (CH_3_); 1652 (C=O). Anal. Calcd. for C_18_H_22_FeN_2_O: C, 63.92; H, 6.56; N, 8.28. Found: C, 63.54; H, 6.37; N 8.28.

*(E)-N'-(4-Nitrobenzylidene)cyclohexanecarbohydrazide* (LASSBio-1603, **20**): LASSBio-1603 (**20**) was obtained in 83% yield as white crystals by condensation of cyclohexane-carbohydrazide (**28**) with 4-nitrobenzaldehyde, m.p. 205–206 °C. ^1^H-NMR (200 MHz) DMSO-*d*_6_ (ppm): 11.59 and 11.17 (s, 1H, CONH), 8.29 (d, 2H, *J* = 8 Hz), 8.07 (s, 1H, N=CH), 7.94 (d, 2H, *J* = 8 Hz), 3.10 and 2.19 (m, 1H), 1.72 (m, 5H, cyclohexyl), 1.32 (m, 5H, cyclohexyl). ^13^C-NMR (50 MHz) DMSO-*d*_6_ (ppm): 177.82 and 172.61 (NC=O), 148.33 (1C), 143.99 (N=CH), 141.03 (1C), 128.33 (2C), 124.63 (2C), 43.42 (1C), 29.47 (2C), 25.89 (3C). IR (KBr) ν_max_ (cm^−1^): 3186 (NH); 2930 (CH_3_); 1668 (C=O) 1520, 1336 (NO_2_). Anal. Calcd. for C_14_H_17_N_3_O_3_: C, 61.08; H, 6.22; N, 15.28. Found: C, 60.81; H, 6.10; N. 14.97.

*(E)-4-((2-(Cyclohexanecarbonyl)hydrazono)methyl)benzoic acid* (LASSBio-1516, **21**): LASSBio-1516 (**21**) was obtained in 83% yield as a white solid by condensation of cyclohexane-carbohydrazide (**28**) with 4-formylbenzoic acid, m.p. > 220 °C. ^1^H-NMR (200 MHz) DMSO-*d*_6_ (ppm): 11.37 and 11.17 (s, 1H, CONH), 8.22 and 8.03 (s, 1H, N=CH), 7.97 (d, 2H, *J* = 8 Hz), 7.76 (d, 2H, *J* = 8 Hz), 3.39 (s, OH), 3.10 and 2.19 (m, 1H), 1.72 (m, 5H, cyclohexyl), 1.32 (m, 5H, cyclohexyl). ^13^C- NMR (50 MHz) DMSO-*d*_6_ (ppm): 177.65 and 172.42 (NC=O), 167.45 (COOH), 145.24 and 141.81 (HC=N), 139.09 (1C), 132.03 (1C), 130.34 (2C), 127.43 (2C), 43.40 (1C), 29.51 (2C), 25.89 (3C). IR (KBr) ν_max_ (cm^−1^): 3211–2855 (OH); 3211 (NH); 2925 (CH_3_); 1669 (C=O). Anal. Calcd. for C_15_H_18_N_2_O_3_: C, 65.68; H, 6.61; N, 10.21. Found: C, 65.46; H, 6.55; N, 9.88.

*(E)-N'-(4-Bromobenzylidene)cyclohexanecarbohydrazide* (LASSBio-1511, **22**): LASSBio-1511 (**22**) was obtained in 98% yield as an orange solid by condensation of cyclohexane-carbohydrazide (**28**) with 4-bromobenzaldehyde, m.p. 215–216 °C. ^1^H-NMR (200 MHz) DMSO-*d*_6_ (ppm): 11.36 and 11.14 (s, 1H, CONH), 8.15 and 7.94 (s, 1H, N=CH), 7.62 (m, 4H,), 3.10 and 2.19 (m, 1H), 1.72 (m, 5H, cyclohexyl), 1.32 (m, 5H, cyclohexyl). ^13^C-NMR (50 MHz) DMSO-*d*_6_ (ppm): 177.51 and 172.29 (NC=O), 145.19 and 141.69 (HC=N), 134.31 (1C), 132.29 (2C), 129.29 (2C), 123.52 (1C), 43.40 (1C), 29.51 (2C), 25.89 (2C), 18.92 (1C). IR (KBr) ν_max_ (cm^−1^): 3188 (NH); 2925 (CH_3_); 1662 (C=O); 1601(C=N); 1138 (Ar-Br). Anal. Calcd. for C_14_H_17_BrN_2_O: C, 54.58; H, 5.54; N, 9.06. Found: C, 54.39; H, 5.50; N 8.92.

*(E)-N'-Benzylidenecyclohexanecarbohydrazide* (LASSBio-1601, **23**): LASSBio-1601 (**23**) was obtained in 99% yield as a white solid by condensation of cyclohexane-carbohydrazide (**28**) with benzaldehyde, m.p. 165 °C. The compound’s data was in agreement with previous reports [36]. ^1^H-NMR (200 MHz) DMSO-*d*_6_ (ppm): 11.28 and 11.08 (s, 1H, CONH), 8.18 and 7.98 (s, 1H, HC=N), 7.66 (m, 2H), 7.43 (m, 3H), 3.10 and 2.19 (m, 1H), 1.72 (m, 5H, cyclohexyl), 1.32 (m, 5H, cyclohexyl). ^13^C-NMR (50 MHz) DMSO-*d*_6_ (ppm): 176.92 and 171.68 (NC=O), 145.90 and 142.28 (HC=N), 135.72 (1C), 129.79 (1C), 128.84 (2C), 126.89 (2C), 43.88 (1C), 29.01 (2C), 25.66 (3C). IR (KBr) ν_max_ (cm^−1^): 3177 (NH); 2927 (CH_3_); 1663 (C=O); 1610 (C=N); 792 and 684 (Aryl). Anal. Calcd. for C_14_H_18_N_2_O: C, 63.92; H, 6.56; N, 8.28. Found: C, 63.44; H, 6.37; N, 8.28.

*(E)-N'-(4-Isopropylbenzylidene)cyclohexanecarbohydrazide* (LASSBio-1508, **24**): LASSBio-1508 (**24**) was obtained in 98% yield as a yellow solid by condensation of cyclohexane-carbohydrazide (**28**) with 4-isopropylbenzaldehyde, m.p. 155–156 °C. ^1^H-NMR (200 MHz) DMSO-*d*_6_ (ppm): 11.22 and 11.03 (s, 1H, CONH), 8.17 and 7.94 (s, 1H, N=CH), 7.56 (d, 2H, *J* = 8 Hz), 7.29 (d, 2H, *J* = 8 Hz), 3.10 and 2.19 (m, 1H), 2.90 (m, 1H), 1.72 (m, 5H, cyclohexyl), 1.32 (m, 5H, cyclohexyl), 1.20 (s, 6H). ^13^C-NMR (50 MHz) DMSO-*d*_6_ (ppm): 177.35 and 172.10 (NC=O), 150.90 and 150.71 (1C), 146.47 and 142.82 (HC=N), 132.70 (1C), 127.52 and 127.33 (2C), 127.25 and 127.25 (2C), 43.42 (1C), 33.88 (1C), 29.57 and 28.92 (2C), 25.88 (3C), 24.20 (2C). IR (KBr) ν_max_ (cm^−1^): 3231 (NH); 2930 (CH_3_); 1662 (C=O); 1601 (C=N). Anal. Calcd. for C_17_H_24_N_2_O:C, 74.96; H, 8.88; N, 10.30. Found: C, 74.82; H, 8.80; N, 10.14.

*(E)-N'-(4-Methoxybenzylidene)cyclohexanecarbohydrazide* (LASSBio-1510, **25**): LASSBio-1510 (**25**) was obtained in 99% yield as a white solid by condensation of cyclohexane-carbohydrazide (**28**) with 4-methoxybenzaldehyde, m.p. 161–162 °C. The compound’s data was in agreement with previous reports [36]. ^1^H-NMR (200 MHz) DMSO-*d*_6_ (ppm): 11.15 and 10.96 (s, 1H, CONH), 8.11 and 7.92 (s, 1H, N=CH), 7.59 (d, 2H, *J* = 8 Hz), 7.02 (d, 2H, *J* = 8 Hz), 3.68 (s, 3H, CH_3_), 3.10 and 2.19 (m,1H), 1.72 (m, 5H, cyclohexyl), 1.32 (m, 5H, cyclohexyl). ^13^C-NMR (50 MHz) DMSO-*d*_6_ (ppm): 176.60 and 171.35 (NC=O), 160.56 (1C), 145.71 and 142.06 (HC=N), 129.90 (1C), 128.37 (2C), 114.27 (2C), 55.19 (1C), 42.80 (1C), 28.97 (2C), 25,27 (3C). IR (KBr) ν_max_ (cm^−1^): 3184 (NH); 2929 (CH_3_); 1654 (C=O); 1608 (C=N), 1252, 1202 (C-O). Anal. Calcd. for C_15_H_20_N_2_O_2_: C, 69.20; H, 7.74; N, 10.76. Found: C, 69.23; H, 7.54; N, 10.46.

*(E)-N'-(4-(Dimethylamino)benzylidene)cyclohexanecarbohydrazide* (LASSBio-1512, **26**): LASSBio-1512 (**26**) was obtained in 89% yield as an orange solid by condensation of cyclohexane-carbohydrazide (**28**) with 4-dimethylaminobenzaldehyde, m.p. 191 °C–192 °C. ^1^H-NMR (200 MHz) DMSO-*d*_6_ (ppm): ^1^H RMN (200 MHz) DMSO-*d*_6_ δ (ppm): 11.06 and 11.64 (s,1H, CONH), 7.95 and 7.82 (s, 1H, N=CH), 7.49 (d, 2H, *J* = 8 Hz), 6.69 (d, 2H, *J* = 8 Hz), 2.91(s, 6H, CH_3_), 3.10 and 2.19 (m, 1H), 1.72 (m, 5H, cyclohexyl), 1.32 (m, 5H, cyclohexyl).^13^C-NMR (50 MHz) DMSO-*d*_6_ (ppm): 176.46 and 171.24 (NC=O), 159.84 (1C), 146.81 and 143.16 (HC=N), 129.53 (2C), 121.78 (1C), 112.41 (2C), 43.40 (1C), 40.00 (2C), 29.51 (2C), 25.89 (3C). IR (KBr) ν_max_ (cm^−1^): 3222 (NH); 2926 (CH_3_); 1658 (C=O); 1607 (C=N); 1362 (Ar-N(CH_3_)_2_). Anal. Calcd. for C_16_H_23_N_3_O: C, 70.30; H, 8.48; N, 15.37. Found: C, 70.44; H, 8.35; N, 15.70.

### 3.2. X-ray Crystallography

In this work, X-ray powder diffraction data and a simulated annealing algorithm implemented in the DASH software program [37] were used to determine the crystal structure of LASSBio-1515, on the basis of previous procedures [38,39]. Thus, a Rietveld refinement of the final crystal structure was conducted using the *Topas Academic* v. 5 software [40]. Data pertaining to the X-ray crystallographic determination of LASSBio-1515 have been deposited in the Cambridge Crystallographic Data Centre with CCDC ID: 929463. These data can be obtained free of charge from the Cambridge Crystallographic Data Centre via www.ccdc.cam.ac.uk/data_request/cif.

### 3.3. Binding Assay

A binding assay was performed between NAH compounds (10 μM or 30 μM) and A_2A_ receptors of rats [41]. [^3^H]CGS21680 (10 nM) was used as A_2A_ agonist radioligand and 30 µM NECA was used to estimate the nonspecific binding. The data were expressed as percent inhibition of control specific binding using the equation: % inhibition =100 − [(measured specific binding/control specific binding) ×100].

### 3.4. Antinociceptive and Anti-Inflammatory Pharmacological Evaluation

#### 3.4.1. Animals

Swiss mice weighing 20–30 g (from the BIOCEN-UFAL) were housed in group cages and maintained on a 12 h light/12 h dark cycle. Animals had free access to food and water at all times. Experiments were carried out according to a protocol approved by the Animal Welfare Committee of Federal University of Alagoas (UFAL) (Number: 026681/2009-23), and in according with the ethical guidelines for investigation of experimental pain in conscious animals.

#### 3.4.2. Reagents

Acetic acid (Merck, Rio de Janeiro, Brasil), arabic gum (Sigma-Aldrich, Rio de Janeiro, RJ, Brasil), morphine sulphate (Dimorf-Cristalia-BR, São Paulo, Brasil), dipyrone (Sigma-Aldrich) and indomethacin (Sigma Aldrich) were obtained from commercial sources. A solution of formalin 2.5% was prepared with formaldehyde (Merck) in saline (NaCl 0.9%).

#### 3.4.3. Acetic Acid-Induced Writhing Test

This test was performed as described [27]. Acetic acid (0.6%, *v*/*v*) was administered i.p. in a volume of 0.1 mL/10 g. The number of writhes, a response consisting of contraction of an abdominal wall, pelvic rotation followed by hind limb extension, was counted during continuous observation for 20 min beginning from 5 min after the acetic acid injection. Dipyrone and compounds (all 100 µmol/kg, oral administration) were administered 60 min before the acetic acid injection. Antinociceptive activity was expressed as inhibition percent of the usual number of writhing observed in control animals. 

#### 3.4.4. Formalin-Induced Nociception

The procedure used was essentially the same as that described previously [26]. Animals received 20 mL of 2.5% formalin solution (0.92% formaldehyde in saline) in the ventral surface of the right hind paw. Animals were observed from 0 to 5 min (neurogenic phase) and from 15 to 30 min (inflammatory phase) and the time that they spent licking the injected paw was recorded and considered as indicative of nociception. Animals received indomethacin or compounds (100 µmol/kg, oral administration) 40 min beforehand. Control animals received vehicle (Arabic gum).

#### 3.4.5. Hot-Plate Test

Mice were treated according to the method described by Kuraishi *et al.* [42]. Animals (*n* = 6) were placed on a hot-plate set at 55 ± 1 °C. Reaction time was recorded when the animals licked their fore and hind-paws and jumped at 30, 60, 90 and 120 min after oral administration of 100 µmol/kg of compounds or reference drug (morphine, 15 µmol/kg. i.p.). Baseline was considered as the mean of reaction time obtained at 30 and 60 min before administration of derivatives or morphine and was defined as normal reaction of animal to the temperature.

#### 3.4.6. Carrageenan-Induced Peritonitis

Peritoneal inflammation was induced according to the method described by Ferrandiz and Alcaraz [28]. A solution of carrageenan 1% (Sigma-Aldrich) was prepared in saline (NaCl 0.9%) and injected into the peritoneal cavity of mice (250 µL/animal). Four h after injection of carrageenan, the animals were killed by cervical dislocation and the peritoneal cavity was washed with 3 mL of cold Hank’s. Compounds and indomethacin were administered at the dose of 100 µmol/kg (p.o.), 30 min before carrageenan injection. Control group received 10 mL/kg of vehicle (Arabic gum, p.o.). The number of cells was quantified by optical microscope, using 100 × lens.

#### 3.4.7. Statistical Analysis

Data obtained from animal experiments are represented by mean ± standard error of the mean (Mean ± S.E.M.). Statistical differences between the treated and the control groups were evaluated by ANOVA in the Prisma^®^ tutorial. Values were considered significant if * *p* < 0.05 and ** *p* < 0.01.

### 3.5. Thermal Sensitization and Mechanical Allodynia Induced by Spinal Nerve Ligation

#### 3.5.1. Animals

Male Wistar rats (*Rattus norvegicus*) weighing 180 to 220 g were used at the start of surgery. The animals were maintained in a temperature (25 °C) and humidity (50%–60%) controlled room on a 12 h light: 12 h dark cycle. The Institutional Animal Care and Use Committee at Universidade Federal do Rio de Janeiro (UFRJ) approved the procedures in this study. Animals were acclimated to the laboratory for at least 30 min before experimental initiation. The study was approved by the Animal Care and Use Committee at Universidade Federal do Rio de Janeiro (UFRJ; Rio de Janeiro, Brazil)

#### 3.5.2. Surgery

Peripheral neuropathy was induced in rats by spinal nerve ligation [33]. Briefly, rats were anesthetized with ketamine (100 mg·kg^−1^ i.p.) and xylazine (5 mg·kg^−1^ i.p.), the skin was sterilized with 0.5% chlorhexidine. A small incision to the skin overlying L5-S1 was made followed by retraction of the paravertebral musculature from the vertebral transverse processes. The L6 transverse process was partially removed, exposing the L4 and L5 spinal nerve. The L5 spinal nerve was tightly ligated using 6-0 silk suture. For sham animals (animals false operated), the same process was carried out, but the spinal nerve was not ligated.

#### 3.5.3. Behavioral Tests

The withdrawal latency was assessed by applying a radiant heat source to the hind paw of the animals [43]. The light beam was interrupted when the animal lifted the hind paw, allowing measurement the time of paw remained in contact with the heat source. Withdrawal latency was determined using a Dynamic Plantar Anesthesiometer (model 37450, UgoBasile SRL, Varese, Italy). The latency to evoke paw-withdrawal was determined with a cut-off value of 30 s. Control latency was determined using the average response of three measurements. Withdrawal threshold to pressure applied in the hind paw, expressed in grams, was measured using a digital analgesic meter (model EFF301, Insight, Sao Paulo, Brazil) [44]. The stimulation of the paw was repeated five times. Withdrawal threshold was determined with a cut-off value of 120 g to avoid potential tissue injury in the absence of response.

#### 3.5.4. Experimental Design

Forty two Wistar rats were randomly divided into sham and spinal nerve ligation (SNL) groups, which were subdivided into six groups: SNL/DMSO, SHAM/DMSO, SNL/(**13**), SNL/(**17**), SNL/(**19**) and SNL/tramadol. At seven days post-surgery, SNL animals were treated by oral administration with compounds during seven or fourteen days. Thermal hyperalgesia and mechanical allodynia were measured at the 10th and 14th day after surgery. In other group, thermal hyperalgesia and mechanical allodynia were measured at the 10th, 14th, 17th and 21th days after surgery.

#### 3.5.5. Statistical Analysis 

Values are expressed as means ± standard error of means (SEM) and one-way ANOVA followed by the Newman–Keuls test for multiple comparisons, using GraphPad Prism (version 5.0; GraphPad Software, Inc., San Diego, CA, USA). *p* values of <0.05 were considered significant.

## 4. Conclusions

A series of cyclohexyl-*N*-acylhydrazones were designed as simplified analogues of the prototype LASSBio-294, synthesized and evaluated as analgesic and anti-inflammatory agents. The configuration and conformation of the privileged structure *N*-acylhydrazone (NAH) were determined from compound **10**. Employing X-ray powder diffraction, the configuration *E* (N=CH) and the antiperiplanar conformation of the amide subunit (-CONH-) of the NAH framework were unequivocally determined. These compounds **10**–**26** and their conformational restricted analogue **9** showed great anti-inflammatory and/or analgesic activity, with compound **13** (LASSBio-1514) standing out due to its important analgesic activity in a neuropathic pain model.
